# Erythrocyte membrane lipidomic signatures in canine lymphoma: immunophenotype-specific alterations and prognostic associations

**DOI:** 10.3389/fvets.2026.1950771

**Published:** 2026-09-24

**Authors:** Valentina Rinaldi, Benedetta Belà, Annamaria Porreca, Isa Fusaro, Alessia Luciani, Gianpaolo Scipioni, Carla Ferreri, Alessandro Gramenzi, Andrea Boari, Paolo Emidio Crisi

**Affiliations:** 1 Department of Veterinary Medicine, University of Teramo, Teramo, Italy; 2 Department of Human Sciences and Promotion of the Quality of Life, San Raffaele, Rome, Italy; 3 Clinical and Molecular Epidemiology, Istituto di Ricovero e Cura a Carattere Scientifico (IRCCS) San Raffaele Roma, Rome, Italy; 4 ISOF, Institute for Organic Synthesis and Photoreactivity, Consiglio Nazionale delle Ricerche, Bologna, Italy

**Keywords:** dog, immunophenotype, lipid, lymphoma, tumor

## Abstract

Lipid metabolic dysregulation is increasingly recognized as a hallmark of cancer. Erythrocyte membrane fatty-acid (FA) profiling may reflect systemic metabolic alterations and provide non-invasive biomarkers of tumor biology. This prospective study investigated the erythrocyte membrane lipidomic profile of dogs with lymphoma and its potential prognostic relevance. Thirty-nine dogs with newly diagnosed lymphoma were enrolled. Clinical staging, flow-cytometric immunophenotyping, and erythrocyte membrane FA analysis were performed at diagnosis. Individual FAs, FA classes, and lipid-derived indices were compared between B-cell and T-cell lymphomas. Lymphoma-specific survival (LSS) was evaluated using Kaplan–Meier analysis and exploratory univariate Cox proportional hazards models. Twenty-five dogs had B-cell lymphoma, and 14 had T-cell lymphoma. T-cell lymphomas were more frequently extranodal, whereas B-cell lymphomas were predominantly multicentric. Palmitoleic acid was significantly lower in T-cell than in B-cell lymphoma. Correlation analysis revealed distinct lipidomic interaction patterns in dogs with lymphoma compared with healthy controls, particularly involving polyunsaturated fatty acids, omega-6 fatty acids, arachidonic acid, and the unsaturation index. *The estimated LSS probability was approximately 50% at 180 days. Exploratory Cox analyses identified nominal associations between mixed diet and a lower hazard of lymphoma-related death, higher oleic acid concentrations and a lower hazard, and higher cis-vaccenic acid concentrations and a higher hazard*. These findings should be interpreted with caution given the limited sample size, multiple comparisons, and lack of multivariable adjustment. Overall, erythrocyte membrane FA profiling identified immunophenotype-related differences and exploratory associations with outcome, supporting further investigation of lipidomics as a potential prognostic tool in canine lymphoma.

## Introduction

Lipid metabolic dysregulation is recognized as a hallmark of cancer (1, 25). In recent years, increasing attention has been directed toward the application of lipidomics in oncology, with the aim of identifying cancer-specific lipid signatures associated with tumor biology and prognosis (26), but also for understanding the connections between the disease and the quality of nutritional intakes (25, 27). Alterations in lipid metabolism may reflect important biological characteristics of cancer cells, including proliferation, aggressiveness, and adaptation to metabolic stress (28). Cancer cells are characterized by enhanced *de novo* fatty-acid (FA) synthesis, leading to increased incorporation of saturated and monounsaturated fatty acids (SFA and MUFA) into cellular membranes, a process that may contribute to resistance against oxidative stress (29). Furthermore, under adverse microenvironmental conditions, including hypoxia and nutrient deprivation, neoplastic cells can accumulate intracellular lipid droplets containing FA, triglycerides, and cholesterol esters, thereby promoting survival during energy stress (30, 31). Because lipid composition may mirror the metabolic and biological behavior of tumors, lipidomic profiling has emerged as a promising source of novel biomarkers that could complement conventional diagnostic and prognostic tools (26). The FA composition of the red blood cell (RBC) membrane reflects the overall distribution of FA families and the physiological status of other body tissues. Since it is influenced by genetic, metabolic, and dietary factors, the RBC membrane FA profile represents a comprehensive biomarker of an individual's homeostatic condition (32). In veterinary medicine, erythrocyte membrane lipidomics has recently been applied to companion animals, providing reference FA profiles in healthy dogs and identifying disease-associated alterations in dogs with chronic enteropathy and cats with chronic enteropathy or small-cell lymphoma (33–36). These findings support the potential utility of erythrocyte membrane fatty acid profiling as a non-invasive biomarker of disease-associated metabolic alterations. Similarly, studies in human oncology have investigated the role of lipid alterations in cancer development, identifying the RBC membrane as a potential biomarker in several neoplastic diseases, including breast, prostate, liver, lung, and colorectal cancers (32, 37–39). In human oncology, a recent study (40) correlated lipid metabolism reprogramming in diffuse large B-cell lymphoma (DLBCL) with disease progression, therapeutic resistance, and immune evasion. Specifically, dysregulated FA, cholesterol, and phospholipid metabolism appeared to promote tumor proliferation, suppress ferroptosis, and modulate the tumor immune microenvironment. Canine lymphoma is the most common hematopoietic neoplasm in dogs, and DLBCL is the most frequently diagnosed subtype (41, 42). This tumor is a highly aggressive malignancy originating from mature B cells and exhibits marked similarities with its human counterpart (43). Several prognostic factors have been investigated, among which immunophenotype, clinical stage, tumor grade, and response to chemotherapy are considered the most clinically relevant (1). The aim of this study was to evaluate the erythrocyte membrane lipidomic profile in dogs with lymphoma and to investigate its potential as a prognostic biomarker.

## Material and methods

### Study population

This prospective longitudinal observational study enrolled dogs presented to the Veterinary Teaching Hospital (VTH), Department of Veterinary Medicine, University of Teramo, Italy, between April 2024 and April 2025. Eligible cases consisted of dogs with newly diagnosed lymphoma, while a small group of healthy dogs was included as a descriptive reference group for baseline lipidomic profiling. Inclusion required the absence of concurrent disease and completion of a standardized diagnostic protocol consisting of: (2) physical examination, complete blood count (CBC), and serum biochemistry; (1) complete staging with thoracic radiographs, abdominal ultrasonography, and fine-needle aspiration (FNA) of the liver and spleen. For dogs with lymphoma, only cases without evident ultrasonographic splenic abnormalities (e.g., honeycomb spleen appearance) were considered eligible; (3) flow cytometric immunophenotyping of lymph node aspirates and bone marrow specimens. Clinical stage was determined according to the World Health Organization (WHO) classification Owen, L.N. (44) Veterinary Public Health Unit and WHO Collaborating Center for Comparative Oncology., 1980); and all dogs received a 15-week CHOP chemotherapy protocol, consisting of cyclophosphamide, doxorubicin, vincristine, and prednisone, as first-line treatment (3). Dogs that had received corticosteroids and/or chemotherapy within the month preceding blood collection were not eligible for enrollment. Dietary information was recorded for all enrolled dogs and classified as home-prepared, exclusively commercial, or mixed (commercial and home-prepared) diet. The diet followed by each dog before the oncologic consultation was not modified for the purpose of the study; the investigators only recorded the dietary regimen as reported by the owners. Dogs with a normal body condition score (BCS) of 5/9, presented at VTH for routine check-up, pre-anesthetic evaluation before neutering or for blood donors' selection were included in the healthy reference group. These patients had no clinical or pathological evidence of disease according to an unremarkable history, physical examination, and results of CBC, serum biochemistry, and voided urine analysis. No medical treatment had been administered to this group of dogs in the past 6 months, except for regular treatments for control of ectoparasites and/or vaccination. The study was performed with full informed consent from the owners and has been approved by the Animal Research and Ethics Committees of the Universities in Chieti-Pescara and Teramo, and by the Experimental Zooprophylactic Institute of AeM (CEISA; UNICHD12 N. 1168).

### Lipidomic analysis

Erythrocytes were isolated from 1 mL of fresh EDTA-anticoagulated whole blood by two consecutive centrifugation steps (3,000 g × 5 min each), followed by complete plasma removal. The packed red blood cells were subsequently washed twice with 0.5 mL of phosphate buffer, centrifuged after each wash (3,000 g × 5 min), and the supernatants were discarded. Erythrocyte lysis was achieved by two successive treatments with 1 mL distilled water. Following centrifugation at 15,000 g × 15 min, the aqueous phase was removed to obtain the erythrocyte membrane pellet. Membrane lipids were then extracted according to the Folch method by adding a 2:1 (v/v) chloroform mixture (2 mL) and subsequently partitioning with 1 mL water. The organic phase containing membrane lipids was collected and evaporated to dryness under reduced pressure. Purified phospholipids were then converted into fatty acid methyl esters (FAMEs) through transesterification with 0.5 M KOH in methanol at room temperature for 10 min. This procedure minimizes oxidation and degradation phenomena while providing an accurate representation of the fatty acid composition of membrane glycerophospholipids, as previously validated using appropriate MUFA and PUFA internal standards. FAMEs were recovered by extraction with n-hexane (2 mL) and water (0.5 mL), and the organic fraction was evaporated to dryness under vacuum. Prior to sample analysis, commercially available standards corresponding to the 10 fatty acids included in the analytical panel (Table 1) were analyzed under identical chromatographic conditions to generate calibration curves for quantitative determination. The resulting FAME extracts were reconstituted in 20 μL n-hexane, and 1 μL was injected into an Agilent 8890 Gas Chromatograph (Agilent Technologies, Milan, Italy) equipped with a flame ionization detector (FID) and a DB-23 capillary column (60 m × 0.25 mm i.d., 0.25 μm film thickness; Agilent Technologies). Chromatographic separation was performed with an initial oven temperature of 165 C maintained for 3 min, followed by a temperature ramp of 1 C/min up to 195 C and a holding period of 40 min. Subsequently, the temperature was increased at 10 C/min to 240 C and maintained for an additional 10 min. Hydrogen was used as the carrier gas at a constant pressure of 16.482 psi. Individual FAMEs were identified by comparison of their retention times with those of authentic reference standards. The analytical method achieved complete separation of the 10 target fatty acids without peak overlap. The analyzed fatty acid panel accounted for approximately 97% of the total chromatographic peak area and included two saturated fatty acids (palmitic and stearic acids), three monounsaturated fatty acids (palmitoleic, oleic, and vaccenic acids), three omega-6 polyunsaturated fatty acids (linoleic, dihomo-γ-linolenic, and arachidonic acids), and two omega-3 polyunsaturated fatty acids (eicosapentaenoic and docosahexaenoic acids). Quantitative concentrations of individual fatty acids were calculated from the corresponding calibration curves and expressed as μg/mL. These values were used to determine total saturated fatty acids (SFA), monounsaturated fatty acids (MUFA), and polyunsaturated fatty acids (PUFA), as well as the SFA/MUFA and omega-6/omega-3 ratios. In addition, the Unsaturation Index (UI), Peroxidation Index (PI), and PUFA Balance were calculated.

**Table 1:** Fatty acid classes, individual fatty acids and lipid-related indices evaluated in the study.

Fatty acid class or index category	Subclass	Individual fatty acids or indices
SFA		Palmitic acid (C16:0)
SFA		Stearic acid (C18:0)
MUFA		Palmitoleic acid (9c, C16:1)
MUFA		Oleic acid (9c, C18:1)
MUFA		Vaccenic acid (11c, C18:1)
PUFA	ω-6	Linoleic acid (C18:2 ω-6)
PUFA	ω-6	Dihomo-gamma-linolenic acid (C20:3n-6)
PUFA	ω-6	Arachidonic acid (AA, C20:4 ω-6)
PUFA	ω-3	Eicosapentaenoic acid (EPA, C20:5 ω-3)
PUFA	ω-3	Docosahexaenoic acid (DHA, C22:6 ω-3)
FA SUM	Saturated fatty acids	SFA
FA SUM	Monounsaturated fatty acids	MUFA
FA SUM	Polyunsaturated fatty acids	PUFA
FA SUM	ω-6 fatty acids	Total ω-6 fatty acids
FA SUM	ω-3 fatty acids	Total ω-3 fatty acids
FA ratios and indices		ω-6/ω-3 ratio
FA ratios and indices		SFA/MUFA ratio
FA ratios and indices		PUFA balance
FA ratios and indices		ω-3/(ω-3 + ω-6)
FA ratios and indices		Unsaturation index (UI)^*^
FA ratios and indices		Peroxidation index (PI)^*^

SFA, saturated fatty acids; MUFA, monounsaturated fatty acids; PUFA, polyunsaturated fatty acids; FA, fatty acid. ^*^The asterisk identifies calculated lipid indices.

### Statistical analysis

Continuous variables were summarized as median and interquartile range, while categorical variables were reported as absolute frequencies and percentages. Baseline clinical characteristics, dietary variables, anatomical localization, immunophenotype, and FA profiles were described for the overall cohort and compared between B-cell and T-cell lymphoma cases. Between-group comparisons were performed using appropriate nonparametric tests for continuous variables and chi-square or Fisher's exact tests for categorical variables, depending on the data distribution and expected cell counts. Statistical significance was set at *p* < 0.05. Given the exploratory and hypothesis-generating nature of the study, no formal adjustment for multiple comparisons was applied. Accordingly, reported *p*-values should be regarded as nominal and interpreted cautiously. Pairwise associations among individual FA, FA classes, and derived lipid indices were evaluated using Pearson correlation coefficients. Correlation matrices were generated separately for diseased and healthy dogs. Correlation strength and direction were represented using a color scale, with blue indicating positive correlations and red indicating negative correlations. Statistical significance was reported within each cell as not significant for *p* ≥ 0.05, ^*^ for *p* < 0.05, ^**^ for *p* < 0.01, and ^***^ for *p* < 0.001 (Figure 1). Lymphoma-specific survival (LSS) was analyzed using the Kaplan–Meier method. LSS was defined as the time from diagnosis to documented lymphoma-related death. Dogs alive at the last available follow-up were censored at the date of their last clinical evaluation. Lymphoma-specific survival probabilities were plotted over time, and censored observations were indicated on the survival curve. The number of subjects at risk was reported below the plot. The association between clinical, dietary, and FA-related variables and LSS was assessed using univariate Cox proportional hazards regression models. Hazard ratios (HRs) with 95% confidence intervals (95% CIs) and *p*-values were reported. HRs greater than 1 were interpreted as indicating an increased hazard of lymphoma-related death, whereas HRs lower than 1 were interpreted as indicating a reduced hazard. Given the limited sample size and the small number of events, survival analyses were performed as univariate exploratory analyses. No multivariable Cox model was fitted to avoid model overfitting and unstable parameter estimates.

**Figure 1:**
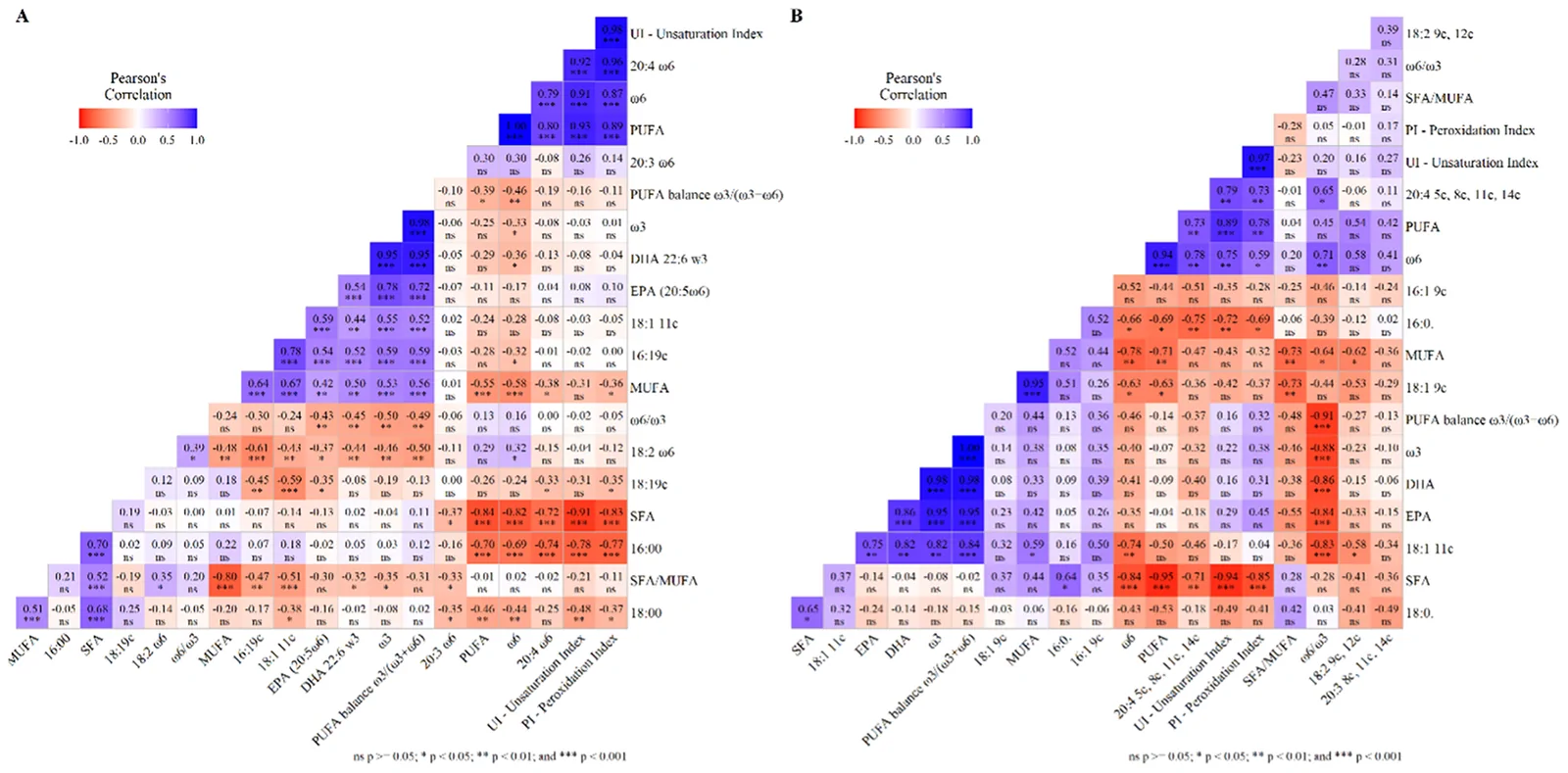
Pearson correlation matrices of fatty-acid composition and lipid indices in diseased dogs and healthy dogs. (A) shows the correlation matrix for dogs with lymphoma, whereas (B) shows the correlation matrix for healthy dogs. Each triangular heatmap reports pairwise Pearson correlation coefficients among individual fatty acids, fatty-acid classes, and derived lipid indices. Blue cells indicate positive correlations, red cells indicate negative correlations, and color intensity reflects the strength of the association. Statistical significance is reported within each cell as follows: not significant for *p* ≥ 0.05, * for *p* < 0.05, ** for *p* < 0.01, and *** for *p* < 0.001.

## Results

### Study population

The study cohort included 39 dogs, of whom 25 (65%) had B-cell lymphoma and 14 (35%) had T-cell lymphoma. The overall median age was 120 months, with an interquartile range of 96.0–144 months. Median body weight was 20.0 kg, with an interquartile range of 10.2–34.8 kg. Most dogs had a BCS < 6 34/39 (87.2%) and were fed a commercial diet 33/39 (84.6%). The distribution of sex did not differ significantly between B-cell and T-cell lymphoma cases (*p*= 0.493). Age was also comparable between groups, with a median of 132 months in B-cell cases and 120 months in T-cell cases (*p*= 0.883). Body weight did not significantly differ between groups (*p*= 0.207), and no significant difference was observed for diet (*p*= 1.000). A difference was observed for BCS. A BCS ≥6 was more frequent among T-cell lymphoma cases, 4 of 14 (28.6%), than among B-cell lymphoma cases, 1 of 25 (4.0%), *p* = 0.047. Given the small number of dogs with BCS ≥6, this finding should be interpreted cautiously. Anatomical localization also differed significantly between groups, *p* = 0.033. Extranodal localization was more frequent in T-cell lymphoma cases, 8 of 14 (57.1%), whereas multicentric localization was more frequent in B-cell lymphoma cases, 20 of 25 (80.0%). Clinical stage according to WHO classification did not differ significantly between B-cell and T-cell cases, *p* = 0.281. For statistical analysis, lymphomas were categorized as multicentric (nodal) or extranodal. Extranodal lymphomas were defined as primary lymphomas arising outside the lymph nodes and included alimentary, hepatosplenic, and other extranodal presentations. Data are summarized in Table 2. The healthy reference group consisted of 12 clinically healthy dogs of different breeds, including mixed-breed dogs (*n* = 5), Golden Retrievers (*n* = 2), Siberian Huskies (*n* = 2), one Labrador Retriever, and one Miniature Poodle. The median age was 100 months (range, 90–156 months), and body weight ranged from 5.0 to 30.0 kg. Both sexes were represented, including intact and neutered animals.

**Table 2:** Clinical and fatty acid-based erythrocyte membrane lipidomic analysis of 39 dogs diagnosed with lymphoma, stratified by immunophenotype (B-cell vs. T-cell).

Variable	Overall (*N* = 39)	B-cell (*n* = 25)	T-cell (*n* = 14)	*p*-value
Sex				0.493
F	2 (5.13%)	1 (4.00%)	1 (7.14%)	
SF	20 (51.3%)	15 (60.0%)	5 (35.7%)	
M	13 (33.3%)	7 (28.0%)	6 (42.9%)	
NM	4 (10.3%)	2 (8.0%)	2 (14.3%)	
Age (months), median [IQR]	120 [96.0–144]	132 [96.0–144]	120 [108–132]	0.883
Body condition score (BCS)				**0.047**
BCS < 6	34 (87.2%)	24 (96.0%)	10 (71.4%)	
BCS ≥ 6	5 (12.8%)	1 (4.0%)	4 (28.6%)	
Weight (kg)	20.0 [10.2–34.8]	18.0 [10.0–30.0]	20.0 [13.0–39.0]	0.207
Diet type				1.000
Commercial	33 (84.6%)	21 (84.0%)	12 (85.7%)	
Mixed	6 (15.4%)	4 (16.0%)	2 (14.3%)	
Anatomical localization				**0.033**
Extranodal	13 (33.3%)	5 (20.0%)	8 (57.1%)	
Multicentric	26 (66.7%)	20 (80.0%)	6 (42.9%)	
WHO clinical stage				0.281
< V	17 (43.6%)	13 (52.0%)	4 (28.6%)	
≥V	22 (56.4%)	12 (48.0%)	10 (71.4%)	
16:0	14.9 [12.3–16.5]	14.6 [12.1–16.4]	15.1 [12.6–19.1]	0.639
16:1 9c	0.72 [0.41–1.06]	0.89 [0.54–1.34]	0.42 [0.36–0.70]	**0.024**
18:0	23.3 [21.1–25.9]	23.3 [21.6–25.8]	23.5 [20.7–28.3]	0.953
18:1 9c	10.6 [8.69–13.3]	10.2 [8.80–13.6]	11.1 [8.94–13.0]	0.884
18:1 11c	3.18 [2.59–4.75]	3.50 [2.92–4.95]	3.04 [2.46–3.52]	0.364
18:2 ω6	12.4 [9.86–13.3]	12.4 [10.7–13.2]	12.4 [9.30–14.2]	0.907
20:3 ω6	1.72 [1.34–2.54]	1.66 [1.28–2.29]	2.18 [1.42–3.23]	0.372
20:4 ω6 (AA)	29.9 [25.5–33.3]	29.9 [28.8–33.0]	28.0 [24.0–34.3]	0.661
EPA (20:5 ω6)	0.15 [0.04–0.26]	0.15 [0.04–0.25]	0.08 [0.04–0.26]	0.395
DHA (22:6 ω3)	0.22 [0.13–0.54]	0.18 [0.13–0.51]	0.22 [0.09–0.54]	0.942
SFA	38.0 [36.2–40.3]	38.0 [36.1–40.3]	38.1 [36.3–41.0]	0.792
MUFA	15.6 [14.1–18.4]	15.6 [14.3–18.7]	15.6 [14.2–16.6]	0.703
PUFA	45.6 [41.1–48.8]	45.4 [41.6–48.3]	46.0 [41.0–49.0]	0.988
?6	44.7 [40.2–48.6]	44.7 [40.7–48.3]	45.2 [40.3–48.8]	0.930
?3	0.43 [0.22–0.70]	0.48 [0.25–0.67]	0.32 [0.17–0.80]	0.529
?6/?3	106 [62.9–215]	103 [64.2–190]	143 [52.5–293]	0.639
SFA/MUFA	2.49 [2.01–2.76]	2.49 [1.95–2.73]	2.52 [2.22–3.11]	0.364
PUFA balance ω3/(ω3 + ω6)	0.94 [0.46–1.56]	0.96 [0.52–1.53]	0.71 [0.35–1.91]	0.629
UI—unsaturation index	172 [157–184]	171 [157–183]	173 [157–187]	0.815
PI—peroxidation index	143 [125–153]	142 [133–153]	146 [121–156]	0.725

Data are presented as median [IQR] or *n* (%). Statistical comparisons were performed using the Mann-Whitney test for continuous variables and chi-square or Fisher's exact tests for categorical variables. F, female; M, Male; SF, spayed female; NM, neutered male; BCS, body condition score; < V, below WHO stage V; >V, WHO clinical stage V;16:0, palmitic acid; 16:1 9c, palmitoleic acid, cis-9; 18:0, stearic acid; 18:1 9c: oleic acid, cis-9; 18:1 11c: vaccenic acid, cis-11; 18:2 ω6: linoleic acid, omega-6; 20:3 ω6: dihomo-gamma-linolenic acid, omega-6; 20:4 ω6: arachidonic acid, omega-6; EPA, eicosapentaenoic acid; DHA, docosahexaenoic acid; SFA, saturated fatty acids; MUFA, monounsaturated fatty acids; PUFA, polyunsaturated fatty acids; ω6, total omega-6 fatty acids; ω3, total omega-3 fatty acids; ω6/ω3, omega-6 to omega-3 ratio; SFA/MUFA, saturated to monounsaturated fatty acid ratio; PUFA balance ω3/(ω3 + ω6): omega-3 balance index, calculated as the proportion of omega-3 fatty acids over the sum of omega-3 and omega-6 fatty acids; UI, unsaturation index; PI, peroxidation index. Bold values indicate statistically significant differences (*p* < 0.05).

### Lipidomic analysis

The erythrocyte membrane fatty acid profile of healthy dogs was characterized by a predominance of polyunsaturated fatty acids (PUFAs), which consistently represented the largest lipid fraction (approximately 48%−61%), followed by saturated fatty acids (SFAs; approximately 28%−37%) and monounsaturated fatty acids (MUFAs; approximately 9%−13%). Omega-6 fatty acids accounted for the majority of the PUFA fraction (approximately 48%−59%), whereas omega-3 fatty acids were present at lower proportions (approximately 1%−4%). Consequently, the ω6/ω3 ratio showed moderate inter-individual variability, ranging from approximately 12–65. The unsaturation index (UI) and peroxidation index (PI) showed relatively limited variability across healthy subjects, with UI values ranging from approximately 187–222 and PI values from 161 to 195, indicating a generally homogeneous degree of membrane unsaturation within the healthy population. Although individual variability was observed for specific fatty acids, the overall lipidomic profile appeared consistent across healthy dogs, without evident outliers.

Regarding FA composition, most individual FAs, FA classes, and derived lipid indices did not significantly differ between B-cell and T-cell lymphoma cases. The only statistically significant difference was observed for 16:1 9c, which was lower in T-cell lymphoma cases than in B-cell lymphoma cases. This result should be considered exploratory, given the number of lipidomic comparisons performed. Median 16:1 9c was 0.42, interquartile range 0.36–0.70, in T-cell cases and 0.89, interquartile range 0.54–1.34, in B-cell cases, *p* = 0.024. This result should be considered exploratory, given the number of lipidomic comparisons performed. No significant differences were found for 16:0, 18:0, 18:1 9c, 18:1 11c, 18:2 ω6, 20:3 ω6, 20:4 ω6, EPA, DHA, SFA, MUFA, PUFA, ω6, ω3, ω6/ω3 ratio, SFA/MUFA ratio, PUFA balance, unsaturation index (UI), or peroxidation index (PI). Kaplan–Meier analysis (Figure 2) showed a progressive reduction in the probability of LSS over the follow-up period. The estimated LSS probability remained close to 100% during the initial observation period and then declined to approximately 50% by around 180 days. The confidence intervals became wider at later time points, reflecting the decreasing number of subjects remaining at risk. Table 3 reports results of the univariate Cox proportional hazards models for LSS. Diet showed a nominal association with LSS, with dogs receiving a mixed diet showing a lower estimated hazard of lymphoma-related death than dogs receiving a commercial diet, HR = 0.11, 95% CI 0.01–0.99, *p* = 0.049. Given the wide confidence interval and the small number of dogs in the mixed-diet group, this result should be regarded as exploratory and interpreted with caution. Among FAs, 18:1 9c was associated with a reduced hazard, HR = 0.73, 95% CI 0.54–0.97, *p* = 0.029, whereas 18:1 11c was associated with an increased hazard, HR = 1.47, 95% CI 1.06–2.03, *p* = 0.020. No statistically significant associations with LSS were observed for age, anatomical localization, immunophenotype, BCS, body weight, 16:00, 16:1 9c, 18:00, 18:2ω6, 20:3ω6, 20:4ω6, EPA, DHA, SFA, MUFA, PUFA, total ω6, total ω3, ω6/ω3 ratio, SFA/MUFA ratio, PUFA balance, UI, or PI. Overall, the results suggest that selected FA, particularly 18:1 9c and 18:1 11c, and diet may be associated with LSS. However, these findings should be interpreted as exploratory given the limited sample size, the number of variables tested, and the absence of multivariable adjustment.

**Figure 2:**
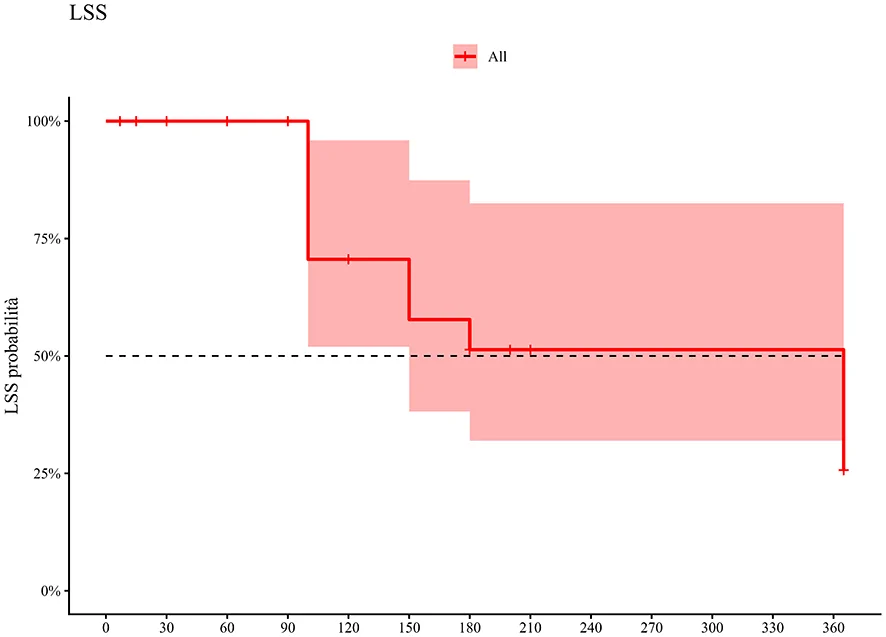
Kaplan–Meier curve for lymphoma-specific survival (LSS) in the overall study population. The red step function represents the estimated LSS probability over time, and the shaded area indicates the 95% confidence interval. Downward steps correspond to lymphoma-related deaths, whereas “+” symbols indicate censored observations. The horizontal dashed line indicates a survival probability of 50%. The number of dogs at risk at each time point is reported below the plot.

**Table 3:** Univariate cox proportional hazards models for lymphoma-specific survival (LSS).

Variables	HR (95%CI)	*p*-value
Age (months)	1.01 (0.99–1.03)	0.353
Anatomical localization (multicentric vs. extranodal)	4.94 (0.59–41.36)	0.141
Immunophenotype (T vs. B)	0.47 (0.11–2.04)	0.315
BCS (≥6 vs. < 6)	1.65 (0.42–6.47)	0.476
Weight	1.00 (0.96–1.04)	0.980
Diet (mixed vs. commercial)	0.11 (0.01–0.99)	0.049
16:0	1.00 (0.76–1.32)	0.991
16:19c	1.52 (0.88–2.62)	0.137
18:0	0.96 (0.79–1.17)	0.702
18:19c	0.73 (0.54–0.97)	0.029
18:1 11c	1.47 (1.06–2.03)	0.020
18:2 ω6	1.24 (0.95–1.62)	0.115
20:3 ω6	0.97 (0.79–1.19)	0.792
20:4 ω6	1.00 (0.82–1.21)	0.961
EPA 20:5ω6	1.97 (0.09–41.90)	0.664
DHA 22:6 ω 3	0.87 (0.15–5.00)	0.875
SFA	0.97 (0.83–1.14)	0.733
MUFA	1.04 (0.92–1.18)	0.510
PUFA	1.04 (0.92–1.17)	0.536
ω6	1.03 (0.29–3.70)	0.963
ω3	0.90 (0.69–1.18)	0.433
ω6/ω3	1.00 (0.99–1.01)	0.943
SFA/MUFA	1.57 (0.34–7.20)	0.559
PUFA balance ω3/(ω3+ω6)	1.01 (0.60–1.68)	0.973
UI—unsaturation Index	1.01 (0.96–1.05)	0.815
PI—peroxidation Index	1.00 (0.95–1.05)	0.890

Hazard ratios (HRs), 95% confidence intervals (95% CIs), and *p*-values are reported for clinical variables, dietary variables, individual fatty acids, fatty-acid classes, and lipid-derived indices. HRs greater than 1 indicate a higher estimated hazard of lymphoma-related death, whereas HRs lower than 1 indicate a lower estimated hazard.

## Discussion

The present study explored the relationship between erythrocyte membrane FA composition, clinicopathological variables, and LSS in dogs with lymphoma, highlighting potential metabolic differences associated with immunophenotype and prognosis. The clinical characteristics of the study population were generally consistent with previous reports on canine lymphoma, particularly the higher prevalence of B-cell lymphoma over T-cell lymphoma and the median age at diagnosis, which closely reflected those reported in the literature (1, 4). An interesting finding of the present study was the association between BCS and lymphoma immunophenotype. Specifically, dogs diagnosed with T-cell lymphoma were more likely to have a BCS ≥6 compared with dogs with B-cell lymphoma. To date, the relationship between BCS and lymphoma immunophenotype has not been extensively investigated in dogs, making direct comparisons with previous studies difficult. Romano and colleagues (5) reported that obesity was not associated with adverse outcomes in dogs with lymphoma, whereas being underweight at the time of diagnosis was associated with shorter survival times. These findings suggest that body condition may be linked to disease biology, although further studies are needed to clarify this relationship. Given the limited number of overweight dogs with T-cell lymphoma included in the present study, this association should be interpreted with caution. The present study also identified a significant association between lymphoma immunophenotype and anatomical localization (*p* = 0.033). B-cell lymphomas were predominantly multicentric, whereas T-cell lymphomas were more frequently associated with extranodal disease. These findings are consistent with previous reports describing distinct anatomical distribution patterns by immunophenotype, with multicentric disease representing the most common presentation of B-cell lymphoma and extranodal involvement occurring more frequently in T-cell lymphoma (1, 6). Most FA and lipid-derived indices did not differ significantly between B-cell and T-cell lymphoma, suggesting that the overall erythrocyte lipidomic profile may be relatively conserved across immunophenotypes. Nevertheless, the lower concentration of palmitoleic acid (16:1n-7) observed in T-cell lymphoma cases may indicate differences in lipid metabolism between lymphoma immunophenotypes. Palmitoleic acid is a MUFA derived from palmitic acid directly transformed by delta-9 desaturase (stearoyl-CoA desaturase, SCD-1), and its increase is connected with the activation of MUFA biosynthesis for membrane fluidity increase in tumors (7). Moreover, its presence in the membrane lipid composition has an impact in cellular signaling and metabolic regulation, and alterations in its abundance have been associated with metabolic reprogramming in several human cancers (8). Therefore, the observed differences may reflect distinct metabolic adaptations between B-cell and T-cell lymphoma subtypes, although the biological significance of this finding remains to be elucidated. The survival analysis identified diet and selected MUFA as potential prognostic variables. It is interesting to note that in a preliminary report on human B-cell lymphoma, similar MUFA biosynthesis activation was detected, with an increase of vaccenic acid (18:1 cis-11), the elongation product of palmitoleic acid (9). These reports reinforce the attention to *de novo* biosynthesis of SFA and MUFA, as a signature of increased cell proliferation, suggesting to deepen the role of nutritional habits to suppress lipogenesis, as it is currently studied in therapeutics (10). Dogs receiving a mixed diet showed a lower estimated hazard of lymphoma-related death compared with dogs fed exclusively commercial diets. Although the mechanisms underlying this association remain speculative, dietary composition may influence systemic metabolism, inflammation, oxidative stress, and membrane lipid composition (11). Nutritional factors are known to influence systemic inflammation, metabolic homeostasis, and immune function in both human and veterinary medicine, and these mechanisms may contribute to cancer progression and treatment response (12, 13). The correlation analysis descriptively suggested different lipidomic association patterns between healthy dogs and dogs with lymphoma. In dogs with lymphoma, strong positive correlations were observed among PUFA, ω6 FA, arachidonic acid (20:4 ω6), and the unsaturation index (UI), suggesting a coordinated pattern of membrane unsaturation and ω6 FA metabolism. Given the central role of arachidonic acid as a precursor of bioactive lipid mediators involved in inflammation, cell proliferation, and tumor progression, these findings may reflect disease-associated alterations in membrane lipid remodeling and inflammatory signaling pathways (1, 14, 24, 25). In contrast, SFA exhibited negative correlations with several measures of unsaturated FA and lipid-derived indices. This inverse relationship is consistent with the dynamic balance between saturated and unsaturated FA within biological membranes and may reflect a relative increase in membrane unsaturation in dogs with lymphoma (14). Such alterations could represent an adaptive response to the metabolic demands of neoplastic cells, which frequently undergo lipid metabolic reprogramming to sustain proliferation, survival, and disease progression (2, 15). Beyond these differences in lipidomic correlations, specific FA were also associated with clinical outcome. Among individual FAs, higher levels of oleic acid (18:1n-9) were associated with longer LSS, whereas increased concentrations of cis-vaccenic acid (18:1 11c) were associated with shorter LSS. The association between higher oleic acid concentrations and improved LSS is of particular interest, suggesting a potential protective role for this FA. Oleic acid has been investigated for its anti-inflammatory and membrane-stabilizing properties (16, 17). Furthermore, several experimental cancer models have demonstrated antitumour effects of oleic acid through suppression of oncogenic signaling pathways, induction of apoptosis, and enhancement of antitumour immune responses (18–20). In addition, inhibition of pathways involved in tumor cell proliferation and migration, including Akt/mTOR and NF-κB signaling, has been reported following oleic acid exposure (21). Although these mechanisms have not been investigated in canine lymphoma, they may provide a biological basis for the favorable association observed in the present study. Another noteworthy finding was the association between higher erythrocyte membrane concentrations of cis-vaccenic acid (18:1 11c) and an increased hazard of lymphoma-related death. Higher concentrations of this FA were associated with shorter LSS, suggesting a potential adverse prognostic significance. Such a marker was also found in human B cell lymphoma, as previously mentioned (9). Although the biological significance of this finding remains to be fully investigated, cis-vaccenic acid is closely linked to endogenous FA synthesis, desaturation, and elongation pathways, particularly through the conversion of palmitoleic acid *via* elongase-mediated reactions (22, 23). Therefore, increased concentrations may reflect alterations in lipid metabolic activity associated with tumor progression rather than a direct detrimental effect of the FA itself (2). The present study has several limitations, including the relatively small sample size and the lack of long-term follow-up data, as the study was completed after a 1-year observation period.

In addition, multiple statistical comparisons were performed without formal multiplicity adjustment because of the exploratory nature of the study. Consequently, an increased risk of Type I error cannot be excluded, and nominally significant findings should be considered hypothesis-generating and require confirmation in larger independent cohorts.

Consequently, the prognostic significance of the observed FA alterations beyond this timeframe remains unknown and should be investigated in future prospective studies. In conclusion, the findings of the present study suggest that alterations in erythrocyte membrane FA composition may reflect biologically relevant metabolic differences among dogs with lymphoma. The associations observed between selected monounsaturated FA and LSS support the hypothesis that lipid metabolism may be associated with clinical outcome. However, given the limited sample size and exploratory nature of the analyses, these findings should be interpreted with caution and require validation in larger prospective studies.

## Data Availability

The original contributions presented in the study are included in the article, further inquiries can be directed to the corresponding author.
